# Optimization of HIV-1 Envelope DNA Vaccine Candidates within Three Different Animal Models, Guinea Pigs, Rabbits and Cynomolgus Macaques 

**DOI:** 10.3390/vaccines1030305

**Published:** 2013-07-19

**Authors:** Marie Borggren, Lasse Vinner, Betina Skovgaard Andresen, Berit Grevstad, Johanna Repits, Mark Melchers, Tara Laura Elvang, Rogier W Sanders, Frédéric Martinon, Nathalie Dereuddre-Bosquet, Emma Joanne Bowles, Guillaume Stewart-Jones, Priscilla Biswas, Gabriella Scarlatti, Marianne Jansson, Leo Heyndrickx, Roger Le Grand, Anders Fomsgaard

**Affiliations:** 1 Department of Microbiology Diagnostics and Virology, Statens Serum Institut, Copenhagen 2300, Denmark; 2 Department of Medical Microbiology, Academic Medical Center of the University of Amsterdam, Amsterdam 1105, The Netherlands; 3 Division of Immuno-Virology, Institute of Emerging Diseases and Immuno Therapies, CEA, Fontenay aux Roses 92260, France; 4 Human Immunology Unit, Weatherall Institute of Molecular Medicine, John Radcliffe Hospital, University of Oxford, Oxford OX3 9DS, UK; 5 San Raffaele Scientific Institute, Milan 20123, Italy; 6 Department of Laboratory Medicine, Lund University, Lund 22184, Sweden; 7 Biomedical Department, Virology Unit, Institute of Tropical Medicine, Antwerp 2000, Belgium; 8 Infectious Disease Research Unit, Clinical Institute, University of Southern Denmark, Odense 5230, Denmark

**Keywords:** DNA vaccine, HIV-1, animal models, envelope, neutralizing antibodies

## Abstract

HIV-1 DNA vaccines have many advantageous features. Evaluation of HIV-1 vaccine candidates often starts in small animal models before macaque and human trials. Here, we selected and optimized DNA vaccine candidates through systematic testing in rabbits for the induction of broadly neutralizing antibodies (bNAb). We compared three different animal models: guinea pigs, rabbits and cynomolgus macaques. Envelope genes from the prototype isolate HIV-1 Bx08 and two elite neutralizers were included. Codon-optimized genes, encoded secreted gp140 or membrane bound gp150, were modified for expression of stabilized soluble trimer gene products, and delivered individually or mixed. Specific IgG after repeated i.d. inoculations with electroporation confirmed *in vivo* expression and immunogenicity. Evaluations of rabbits and guinea pigs displayed similar results. The superior DNA construct in rabbits was a trivalent mix of non-modified codon-optimized gp140 envelope genes. Despite NAb responses with some potency and breadth in guinea pigs and rabbits, the DNA vaccinated macaques displayed less bNAb activity. It was concluded that a trivalent mix of non-modified gp140 genes from rationally selected clinical isolates was, in this study, the best option to induce high and broad NAb in the rabbit model, but this optimization does not directly translate into similar responses in cynomolgus macaques.

## 1. Introduction

The ability to elicit HIV-1 neutralizing antibodies (Nabs) is likely to be an essential feature of protective HIV-1 vaccines. The HIV envelope spike is the only glycoprotein presented on the virion and on the surface of infected cells for antibody binding and neutralization, by broadly neutralizing antibodies (bNAbs). Five areas on the HIV trimeric spike have been identified so far as conserved targets for broadly neutralizing monoclonal antibodies cloned from patients including elite neutralizers [1]. Extensive attempts have been undertaken to construct immunogens and use different vaccine strategies to direct antibodies to these areas and to improve functionality, also encompassing antibody-dependent cell-mediated cytotoxicity (ADCC) [2]. However, the growing knowledge of neutralizing epitope structures on the HIV-1 Env does not automatically translate into the generation of improved immunogens, emphasizing the importance of continuing all approaches in the search for HIV-1 vaccine immunogens. Thus, lessons may still be learned from envelopes of rationally selected and/or modified clinical HIV-1 strains e.g. from patients with bNAbs, ADCC and/or a defined favorable clinical course. 

A stable mimic of the native envelope spike would be an ideal HIV-1 vaccine immunogen, but is technically challenging to construct and produce [3]. Successful attempts to produce *in vitro* stabilized recombinant glycoproteins include the introduction of SOSIP mutations [4,5] and isoleucine-zipper trimerization signals [6,7], combined with improved gp120/gp41 cleavage site [8]. These modifications were also efficient in inducing neutralizing antibodies [9,10,11]. However, a DNA vaccine expressing selected envelopes intracellularly and *in vivo* can potentially more closely mimic the native structure and glycosylations, which may differ from *in vitro* cell line expressed proteins [3]. In addition, a naked DNA vaccine displays the benefits of proven safety, easy manipulation and manufacturing, no anti-vector immunity, and contains in itself an adjuvant effect [12,13]. DNA constructs are also convenient for screening and selection of envelopes which can be rationally modified and tested subsequently to guide protein immunogen production [14]. Despite promising initial studies in small animal models, naked DNA vaccines showed lower immune potency in humans and non-human primates [13]. However, enhanced immunogenicity has now been obtained with several improvements making second generation DNA vaccines ready for trials and use in larger animal models, including humans [15,16,17]. The optimizations of potency include codon-optimized gene sequences [18,19], repeated injection regimens, the inclusion of plasmid adjuvants and various mixed modality (prime-boost) strategies [13,14]. Use of *in vivo* electroporation as a DNA delivery method has proven very effective in enhancing uptake and immunogenicity of DNA vaccines [20,21,22,23]. 

SIV/SHIV infection of macaques is the most reliable animal model for preclinical testing of candidate HIV vaccines. However, before such testing, evaluation of potential immunogen candidates needs to be conducted by screening of several immunogens and improved gene versions in smaller animals, such as rabbits or guinea pigs. The rabbit model (*Oryctolagus cuniculus*) is increasingly used in preclinical HIV-1 vaccine development studies. Firstly, rabbits are large enough to yield sufficient volumes of serum for extensive testing, yet much less challenging to house than non-human primates. Secondly, the rabbit litter size is also large, making it possible to breed for experimental use without endangering the species. The rabbit antibody heavy-chain third complementary-determining region (H3 CDR) is comparable to the length of the VH3 CDRs of human antibodies, whereas the mouse has a shorter VH3 CDR [24,25]. Since length and flexibility in H3 CDRs are structural features necessary for some monoclonal bNAbs [26,27,28,29,30], the rabbit model provides an opportunity for such antibodies to develop. Mice sera are limited in volume and may contain cytostatic factors that down-modulate CD4 receptors on human cells [31], making it less suitable when screening vaccine candidates in HIV NAb assays. Utilizing guinea pigs as models has some of the drawbacks of that with other rodents, but guinea pigs have larger blood volumes than mice and are relatively inexpensive and easy to house and handle.

In this study, we have optimized DNA *env* constructs for immunogenicity, in rabbits and guinea pigs following several steps. The DNA constructs used were based upon the viral reference strain HIV-1_Bx08_, shown to be commonly recognized by immune sera from a variety of patients [32], and thus, exposing common epitopes for NAbs [32]. We have previously shown that the codon-optimized *env*_Bx08_ can induce NAbs with limited breadth [18,33,34]. To select potentially better clinical HIV-1 Env immunogens than the Env_Bx08_, we now hypothesized the opposite, namely that envelope immunogens, which are instead derived from patients with broad neutralizing activity or elite neutralizers, may potentially induce antibodies of broader neutralizing nature. To test this hypothesis, two envelope genes were selected this way and developed into DNA vaccine constructs, and used in a trivalent formulation combined with *env*_Bx08_. Furthermore, the immunogenicity of *env* constructs was evaluated with or without the SOSIP-modifications, aiming to stabilize the envelope protein in trimeric conformation. Finally, the optimal vaccine candidate in rabbits and guinea pigs was further tested for immunogenicity in cynomolgus macaques and compared to the immune responses elicited in the smaller animal models.

## 2. Experimental Section

### 2.1. DNA Vaccine Plasmids

The construction of Bx08 gp140 (Genbank JX473289) plasmid used codons from highly expressed human genes as described earlier [18,33,34] and two other primary Envs from Danish patients, ctl21 (JX473290) and ctl27 (JX473291), were similarly codon optimized. Seven different clade B *env* constructs were synthesized (syn.) and used (syn.gp140_Bx08_, syn.gp150_Bx08_, syn.gp140_ctl21_, syn.gp140_ctl27_, syn._gp140Bx08 SOSIP.R6-IZ-H8_, syn.gp140_ctl21 SOSIP.R6-IZ-H8 _or syn.gp140_ctl27 SOSIP.R6-IZ-H8_). We have previously described the construction of synthetic *env*_Bx08_ plasmids encoding secreted gp140 and membrane-bound gp150 from HIV-1 Bx08 [18,33,34]. The two primary *env*_ctl21_ and *env*_ctl27_ were PCR-amplified from isolated patient virus, cloned, sequenced and then synthesized using only codons from highly expressed human genes (completely codon exchanged) [34,35]. All genes were cloned into the previously described mammalian expression vector pPPI4 [8,10,36]. Plasmids encoding SOSIP.R6-IZ-H8 gp140 variants were constructed as follows: Amino acid substitutions (HxB2 numbering) A501C, T605C and I559P (SOSIP) were introduced as previously described [36]. Additionally, the proteolytic gp120/gp41 cleavage site REKR was substituted with a hexa-arginine motif (R6) to increase cleavage [8]. Together these amino acid substitutions are referred to as SOSIP.R6. The isoleucine zipper (IZ) domain was added to the gp140 *C*-terminus to facilitate gp140 trimerization [7,37,38]. Also, eight histidine residues (H8) were added to allow downstream protein purification procedures. The vector expressing the Env proteins has been described elsewhere [39,40], but was further modified by mutagenesis to contain a multiple cloning site, including a *Hin*d III site, between the tPA sequences and the *env* sequences. The *env* sequences used in this study were then sub-cloned into the resulting vector using *Hin*d III and *Bam*H I. Protein expression was controlled with HEK 293T cells grown in DMEM (Gibco, Carlsbad, CA, USA), supplemented with 10% fetal calf serum (FCS), penicillin and streptomycin. Transfection was performed with Polyfect transfection agent (Qiagen, Hilden, Germany) and expressed proteins were separated in an 8% tris-glycin gel (Invitrogen, Carlsbad, CA, USA). Env proteins were detected by Western blotting using human anti-HIV polyclonal antisera and visualized with a goat HRP-conjugated antihuman IgG (Kirkegaard and Perry Laboratories, Gaithersburg, MD, USA). Membranes were developed with SuperSignal West Femto (Pierce, Rockford, IL, USA) and chemiluminiescence was detected with an UVP (AH Diagnostics, Aarhus, Denmark).

### 2.2. Recombinant Glycoprotein (gp)140

rgp140 clade C heterotrimer protein mix was produced by transient transfection of HEK 293 T cells. In short, 2 mg plasmid DNA with multiple clade C *env* expressed constructs, complexed with 3.6 mg PEI, was added to cells. Supernatant was collected after 48 and 96 hours, and after adjusting to pH 8, the media was passed over a cobalt chloride metal-affinity column made from Talon Superflow resin (Clontech, Palo Alto, CA, USA). Protein was eluted with 250 nM imidazole and concentrated and separated by gel filtration chromatography using a Superdex200 26/60 size-exclusion column (GE Healthcare, Buckinghamshire, UK). The gp140 trimer fractions were identified and further purified using a GNA-lectin resin (Vectorlabs, Burlingame, CA, USA).

### 2.3. Animal Immunizations

**Figure 1:**
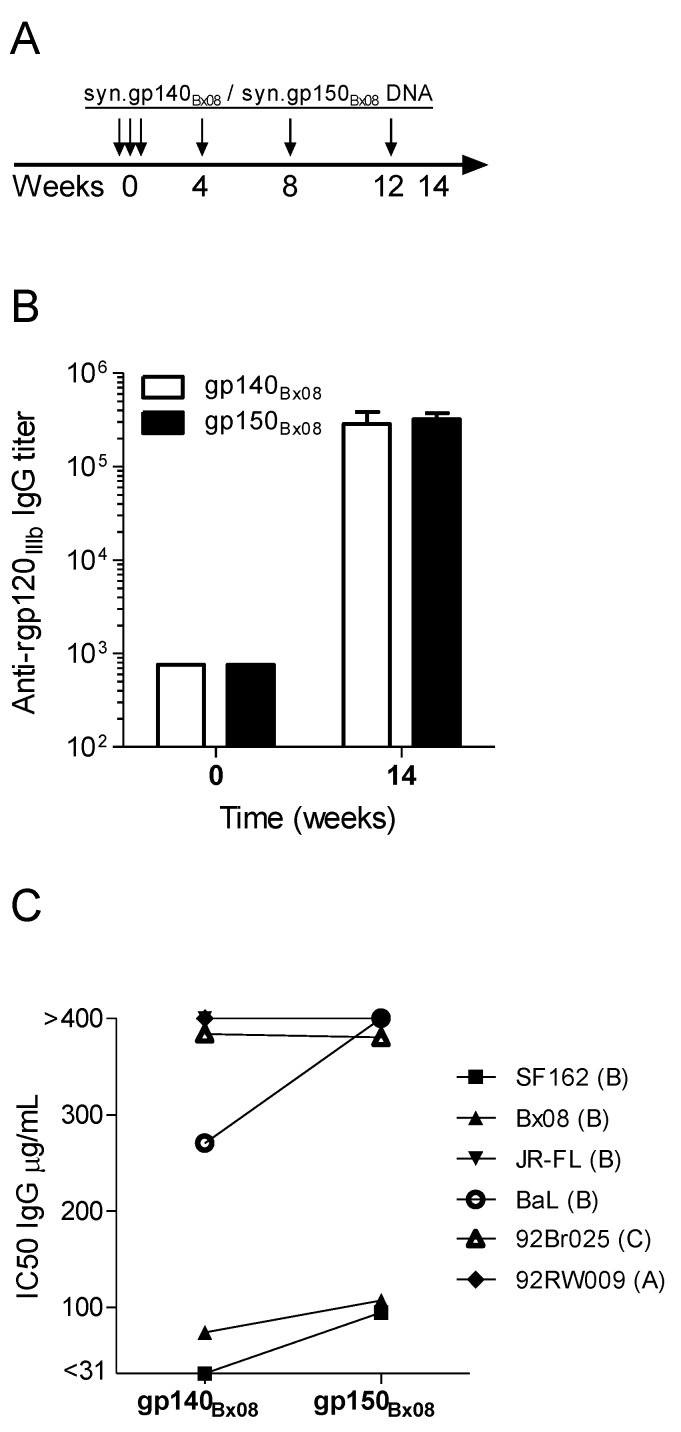
Immunization regimen and comparing antibody responses in syn.gp140_Bx08_ or syn.gp150_Bx08_. DNA vaccinated rabbits. (**A**) Schematic immunization schedule with vertical arrows indicating immunizations. Sera were collected before immunization (w0) and two weeks after last immunization (w14). (**B**) Average IgG response against recombinant gp120_IIIb_ (rgp120_IIIb_) in immunized rabbits (n = 4). (**C**) Average neutralizing activity, expressed as IC50, of purified IgG from week 14 rabbit sera against pseudotype virus strains of clade B, C and A (SF162, Bx08, JR-FL, BaL, 92Br025 and 92RW009).

Ten week old female nulliparous New Zealand white rabbits purchased from Charles River Laboratories were housed at Statens Serum Institute Animal Facility (Copenhagen, Denmark). Acclimatization was at least 10 days prior to any experimental procedures. Animal experiments were performed by certified animal handlers and according to the Animal Experimentation Act of Denmark and European Convention ETS 123. Groups of four rabbits were immunized at week 0 (three times during the first week), 4, 8 and 12 with 200 µg DNA injected intradermal (i.d.) and distributed at two injections sites (Figure 1A). The mode of “intensive” priming within week 0 (3 × 200 µg DNA) was initially compared with single DNA immunization and protein immunization. and demonstrated a more rapid and uniform antibody response than both other immunizations (Supplementary Figure 1). Subsequent electroporation using OncoVet™ system (CytoPulse Sciencies/Cellectis, Romainville, France) was done over each injected area. Four groups of rabbits were used, receiving syn.gp140_Bx08_, syn.gp150_Bx08_, syn.gp140_mix_ (syn.gp140_ctl21_ + syn.gp140_ctl27_ + syn.gp140_Bx08_) or syn._gp140mix modified_ (syn.gp140_Bx08 SOSIP.R6-IZ-H8_ + syn.gp140_ctl21 SOSIP.R6-IZ-H8_ + syn.gp140_ctl27 SOSIP.R6-IZ-H8_). The amount of DNA constructs in the mixed formulations was 1:1:1, giving in total a 200 µg/immunization. In all four groups, blood was collected before immunization (w0) and two weeks after last immunization (w14). In addition, rabbits immunized with syn.gp140_mix_ had blood collected at each re-immunization (w4, 8, 12) and rabbits immunized with syn.gp140_mix__SOSIP.R6-IZ-H8_ had blood collected every week until w6 (w1, 2, 3, 4, 5, 6), then every second week until w14 (w8, 10, 12, 14).

Dunkin Hartley guinea pigs (HsdPoc:DH) were housed at Statens Serum Institute Animal Facility. Acclimatization was at least one week prior to any experimental procedures. Groups with four 12 week old guinea pigs were immunized at week 0, 4, 8 and 12 with 50 µg DNA injected i.d. and localized to either side of the abdomen area. The vaccination area was subsequently electroporated using the OncoVet™ system. Three groups of guinea pigs were used receiving syn.gp140_Bx08_, syn.gp140_mix_ or syn.gp140_mix modified_. Blood samples from a vessel near the eye were taken every second week of the immunization schedule. 

Adult cynomolgus macaques (*Macaca fascicularis*), imported from Mauritius, were housed at the CEA facilities (Fontenay aux Roses, Paris, France) and handled in accordance with French national regulations and under veterinary inspectors (Permit number: A 92-032-02). All procedures were carried out under general anesthesia with intramuscular injection of 10 mg/kg ketamine (Rhône-Mérieux, Lyon, France). Four macaques were immunized by the same regimen as the rabbit protocol with intensive priming at week 0, and followed by three subsequent immunizations at w5, 9 and 13, using 800 µg DNA distributed intradermal at four injection sites, and followed by electroporation. All four animals received the DNA construct syn.gp140_mix_. At w17, the macaques were injected with 80 µg clade C rgp140 heterotrimer protein mixed with 800 µg of the DNA construct syn.gp140_mix_, and no additional adjuvant. Again the injections were followed by electroporation. Blood was collected before immunization (w0) and at different time points for assessing the immune response. 

### 2.4. Anti-Env Antibody ELISA

For assessing specific anti-gp120 IgG three different ELISA assays were established for the three different animal models: rabbits, guinea pigs and cynomolgus macaques. The detailed protocol for the rabbit ELISA is summarized here, followed by minor modifications for the guinea pig and macaque protocols. Maxisorp 96-well plates (Nunc) were coated overnight with recombinant gp120_IIIB_ protein (Fitzgerald Industries International, Concord, USA) in carbonate buffer, pH 9.6. Plates were blocked the following day for 1 h at room temperature with blocking buffer containing PBS, 1% BSA, 10% FCS and 1% Triton X-100. Rabbit sera were subsequently added in serial dilutions, diluted in blocking buffer. After an overnight incubation at room temperature, plates were washed five times with washing buffer (PBS, 0.01% Triton X-100). HRP-conjugated mouse anti-rabbit (Sigma, A1949, St. Louis, MO, USA) antibody was added at a 1/2,000 dilution. After 1 h incubation at room temperature, plates were washed and a one-step TMB substrate (Kem-En-Tec Diagnostics, Copenhagen, Denmark) was added. The colorimetric reaction was stopped with 0.2 M H_2_SO_4_ and absorbance values were read at 540 nm. Titers were defined as the lowest reciprocal dilution yielding an absorbance value greater than the optical density of twice the background absorbance (wells containing blocking buffer). A mixture of pre-defined high-titer rabbit sera was used as positive control [41]. 

The ELISA assay for detection of guinea pig and macaque specific anti-gp120 IgG was modified with a prolonged overnight incubation for the coating step to twice overnight. Blocking buffer in the guinea pig assay was PBS, Tween-20, 5% rabbit-normal-serum and in the macaque assay PBS, 1% BSA, 2% skimmed milk powder, 1% Triton X-100. The blocking step was carried out for 1 h on a shaker. Dilution buffer in the guinea pig assay was PBS, 0.05% Tween-20, and in the macaque assay it was the same as blocking buffer. The overnight incubation with diluted animal sera was carried out on a shaker. The guinea pig assay used HRP-conjugated rabbit anti-guinea pig (Sigma, A5545) antibody at a 1/50,000 dilution and the macaque assay used HRP-conjugated mouse anti-human (BD, 555788, Franklin Lakes, NJ, USA) antibody at a 1/500 dilution. The 1 h incubation with conjugated antibody was carried out on a shaker. The colorimetric reaction was terminated with 1 M H_2_SO_4_. The guinea pig assay used a mixture of high-titer guinea pig serum as a positive control and the macaque assay used IgG purified from pooled HIV-positive patient serum. 

### 2.5. Neutralization Assays and env Selection

Neutralizing activity in sera from immunized animals was analyzed in the pseudovirus-TZMbl assay as described elsewhere [42,43]. Briefly, purified IgG from rabbit sera was used in the TZMbl assay diluted in four 2-fold dilutions, starting at a final concentration of 250 or 400 µg/mL. Rabbit IgG was purified from heat inactivated sera using Protein G HP SpinTrap columns (GE Healthcare). Heat inactivated serial diluted guinea pig and macaque sera were used directly in the TZMbl assay, starting at 1/20 or 1/30 dilution, respectively, and diluted in two-fold steps. Neutralizing activity was expressed as the IgG concentration or reciprocal serum dilution that established 50% inhibition (IC50) of virus infection, as determined by the method described in Fenyö *et al*. in 2009 [44].

The ctl21 and ctl27 *envs* were selected by screening of patient sera or EDTA plasma for neutralization activity, using PHA-P-stimulated donor peripheral blood mononuclear cells (PBMCs), cultured in RPMI 1640 and Glutamax media (Gibco) supplemented with 10% FCS, recombinant human IL-2, penicillin and streptomycin. Briefly, cells were seeded in 96-well tissue culture plates (10^5^/well) and virus, pre-incubated 1 h with heat-inactivated plasma or serum samples, and added to the wells. Infection was allowed for 24 h, then the plate was washed and new medium added. Culture supernatant was harvested at day 3, 4 and 5 and assayed for p24 production [45]. As a negative control, cells and virus were incubated with serum from a non-infected individual. 

### 2.6. Statistical Analysis

Differences in neutralizing activity between groups against various pseudotype viruses were evaluated for statistical significance by a Wilcoxon signed rank test. Two-way ANOVA was used to calculate differences in antibody titers between immunization groups. Comparison of neutralization over time in the macaque group was tested using one-way ANOVA with Dunn´s post test. GraphPad Prism v. 5.0 was used for all analyses.

## 3. Results

### 3.1. Immunogenicity and Heterologous Neutralization Elicited by DNA Vaccine Encoding Secreted gp140Bx08 *versus* Membrane-Bound gp150Bx08 Gene Product

To optimize the DNA vaccine to elicit a high and broad immune response in rabbits, the initial evaluation of *env* DNA concerned the use of gp140 or gp150 genes. The syn.gp150_Bx08_ or syn.gp140_Bx08_ DNA plasmids genes are translated into membrane-bound or secreted glycoproteins, respectively [18,34], and induce an antibody response of equal magnitude in guinea pigs [33,34]. The amount of Env-specific binding IgG in rabbit sera 14 weeks after immunization was assessed against recombinant gp120_IIIb_ (Figure 1). Specific IgG antibody titers were induced in the rabbits by both DNA constructs, with a similar increase of >2 logs over the baseline level. Purified serum IgG from week 14 was analyzed for neutralizing activity against a panel of six different HIV-1 viruses (clades A–C). Results are depicted as 50% inhibitory concentration (IC50) of purified serum IgG (Figure 1C and Supplementary Table 1 for individual IC50 values). No significant difference in neutralizing activity was seen in rabbits by the two different constructs. Three viruses (SF162, Bx08 and BaL) were easier to neutralize with IgG from syn.gp140_Bx08_ rabbit antisera than IgG from syn.gp150_Bx08_ antisera. Thus, both syn.gp140_Bx08_ and syn.gp150_Bx08_ were able to induce a potent antibody response in rabbits demonstrating neutralizing effect on four or three viral strains out of six, respectively, at IgG concentrations between 31 and 400 µg/mL. The viral strains most sensitive to neutralization were all Tier 1 of clade B, which was expected since the DNA construct originated from a clade B virus. Based on this and the possibility of comparing the syn.gp140_Bx08_ with other gp140 constructs, the results from syn.gp140_Bx08_ immunization were included in further analysis.

### 3.2. Neutralization Induced by Trivalent *versus* Monovalent env DNA Vaccines

To broaden the heterologous neutralization capacity induced by syn.gp140 of Bx08, both rabbits and guinea pigs were immunized with a mix of rationally selected HIV *env* genes added to the Bx08 and compared to immunization with monovalent Bx08 immunization. Rational selection of the additional *env* genes was based on screening of neutralizing activity of infected patients’ plasma samples. We hypothesized that envelope immunogens derived from virus of patients with broad neutralizing activity may induce similarly broadly neutralizing antibodies upon immunization in animals or humans if delivered as optimized DNA vaccine constructs. 

Plasma samples (n = 35) from Danish HIV-1-infected treatment-naïve individuals were collected [46] and screened for neutralization against HIV-1 virus isolates, four clade B and one A1D intersubtype recombinant [47] (Table 1). As expected, the sensitivity to neutralization varied among the virus isolates, with clade B HIV-1_BaL_ being most sensitive to neutralization and recombinant A1D HIV-1_DK1_ least sensitive. In many samples, neutralization was primarily directed against one or two viruses, but in 17 sera (49%) the neutralizing effect was detected against all five isolates, including the A1D recombinant. Among these, two plasma samples, ctl21 and ctl27, obtained from a male and a female with 9 and 3.5 years of infection, respectively, displayed robust and balanced neutralization titers against all five viruses. To test the hypothesis, the *env* region including V1-V5 of the clade B virus isolates from ctl21 and ctl27 were cloned, sequenced and synthesized as codon-optimized DNA vaccine constructs, flanked by the *N*- and *C*-terminal region of gp120 and the extracellular part of the gp41 region from the HIV-1_Bx08_ *env* cassette (see Figure 3A and [18]). The constructs were control sequenced and tested for successful *in vitro* expression of functional envelope glycoproteins (CD4 binding) (data not shown).

**Table 1:** HIV-1-specific neutralizing activity in serum from infected individuals (n = 35). Given reciprocal titers correspond to 1/dilution of serum giving 80% inhibitory concentration (IC80) in the PBMC neutralization assay. Color coding: IC80 < 5: no color, 5–25: yellow, 25–125: orange or 125–625: red.

Serum ID	HIV-1 panel (subtype)				
	Bx08 ^§^ (B)	SF162(B)	BaL(B)	JR-FL(B)	DK1 ^#^ (A1D)
ctl31	>625	6	103	14	<5
**ctl21**	**273**	**625**	**158**	**72**	**38**
ctl33	178	59	19	20	<5
**ctl27**	**63**	**254**	**106**	**113**	**19**
ctl47	50	31	>5	66	<5
ctl25	47	11	>625	19	8
ctl38	42	281	438	55	<5
ctl30	42	16	43	39	123
ctl37	32	5	306	22	8
ctl18	29	17	57	11	10
ctl17	27	11	135	13	15
ctl12	23	8	9	6	51
ctl43	18	37	>5	149	8
ctl44	16	6	>5	64	13
ctl40	15	11	>5	64	<5
ctl11	12	13	102	11	24
ctl15	12	13	10	10	11
ctl14	8	>5	36	299	32
ctl23	8	15	>625	11	<5
ctl19	>5	>5	>5	>5	>5
ctl29	<5	12	166	60	13
ctl24	<5	>5	>5	>5	>5
ctl39	>5	15	>5	<5	<5
ctl20	5	>5	>5	<5	<5
ctl46	>5	>5	>5	<5	<5
ctl26	>5	>5	>5	<5	<5
ctl36	>5	<5	12	>5	<5
ctl22	<5	>5	>5	<5	>5
ctl34	>5	<5	>5	<5	<5
ctl45	>5	<5	>5	<5	<5
ctl28	>5	<5	>5	<5	<5
ctl32	>5	<5	<5	<5	<5
ctl35	<5	>5	211	<5	<5
ctl13	<5	>5	>5	<5	<5
ctl16	<5	>5	>5	<5	<5

^§^ Four of the HIV-1 panel isolates are R5 clade B. ^#^ The DK1 isolate is an A1D intersubtype recombinant form with a clade A envelope gene [47].

**Figure 2:**
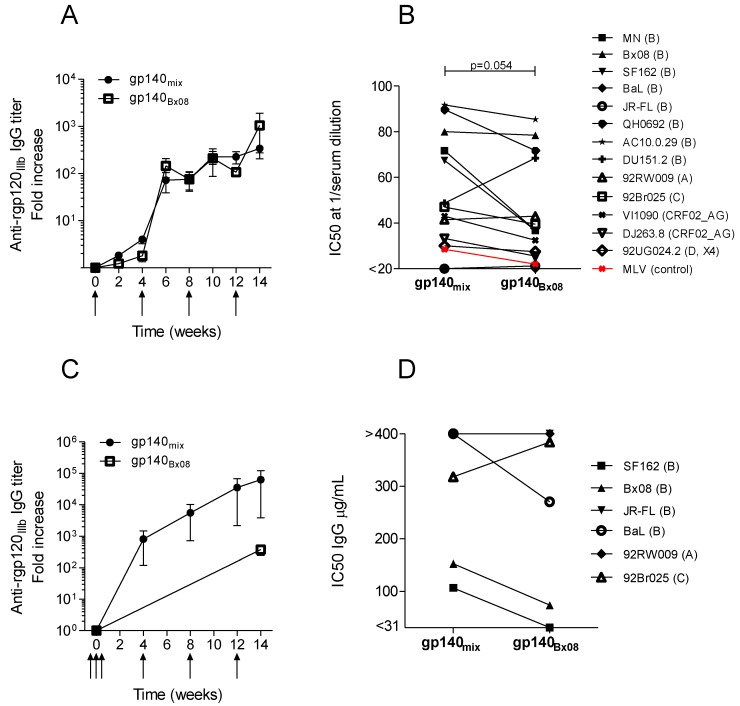
Comparison of the immune responses in animals vaccinated with monovalent or trivalent DNA. Average IgG responses against rgp120_IIIb_ in immunized. (**A**) guinea pigs (n = 4) and (**C**) rabbits (n = 4). Immunization time points are indicated with arrows. Average neutralizing activity, expressed as IC50, of diluted guinea pig serum (**B**) or purified rabbit IgG (**D**) from week 14 against pseudotype virus strains of clade A–D and CRF02_AG. Unrelated MLV pseudotype virus was included as non-specific HIV control in the guinea pig setup (red). IgG titers (**C**) and IC50 values (**D**) from syn.gp140_Bx08_ in the rabbit model are derived from Figure 1B,C.

Guinea pig and rabbit groups were immunized with a trivalent mix encoding syn.gp140_Bx08_, syn.gp140_ctl21_ and syn.gp140_ctl27_ to facilitate heterotrimer formation (referred to as syn.gp140_mix_). Guinea pigs were also immunized with the same single DNA construct, syn.gp140_Bx08_, as used in rabbits in Figure 1. Monovalent and trivalent DNA immunizations demonstrated similar immunogenicity in guinea pigs (Figure 2A). In the rabbit model, the syn.gp140_mix_ induced a higher fold increase in IgG response at w14 than syn.gp140_Bx08_ from Figure 1B (Figure 2C). Immune sera obtained week 14 from guinea pigs and rabbits were analyzed for neutralizing activity (Figure 2B,D, and Supplementary Table 1). Guinea pig sera were diluted and used directly in the TZMbl assay, whereas IgG had to be purified from the rabbit sera because of interference observed in some samples. Guinea pig sera and rabbit IgG were tested for NAbs against a panel of 13 or six different viruses, respectively. In the guinea pig model, syn.gp140_mix_ tended to induce higher NAb titers to most viruses tested (Figure 2B) than monomeric syn.gp140_Bx08_, although this was not statistically significant (*p* = 0.054, Wilcoxon signed rank test). In the rabbit model, this tendency was less pronounced (Figure 2D). For both guinea pig sera and rabbit IgG, there was a large variation in neutralizing activity; however, the clade B viruses were the most sensitive to neutralization. Pseudotype virus expressing the unrelated murine leukemia virus (MLV) envelope was included as controls when testing guinea pigs sera and demonstrated no vaccine-induced unspecific effect (Figure 2B). Taken together, these results tended to favor the trivalent mix although broader neutralization could not be demonstrated in the rabbit model. However, since the trivalent mixture induced somewhat higher and broader neutralization in guinea pigs to most viruses and a somewhat higher cross-reacting antibody titer (anti-gp120_IIIb_) in rabbits, the syn.gp140_mix_ was modified and used in further optimization experiments. In addition, the mixing approach has proven effective in other studies [48,49,50,51].

### 3.3. Trimeric env Modifications of DNA Vaccines Hold Different Immunogenicity in Guinea Pigs and Rabbits

**Figure 3:**
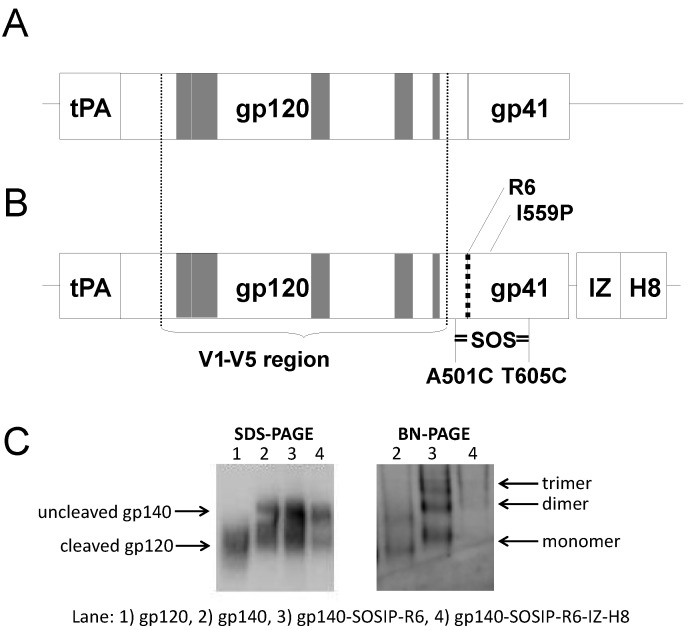
Schematic representation of HIV-1 envelope DNA constructs and protein expression. DNA constructs encoding gp140. The tissue plasminogen-activator leader sequence (tPA) and the region encoding the variable regions V1 to V5 are indicated (grey boxes). (**A**) The gp140_ctl21/27_ construct with V1-V5 region from ctl21 and ctl27 *env* flanked by Bx08 *env*. (**B**) DNA construct encoding modified gp140 including the SOSIP amino acid substitutions A501C, T605C and I559P (SOSIP), the hexa-arginine cleavage site (R6), the introduced isoleucine-zipper motif (IZ) and the histidine tag (H8). (**C**) Western blot analysis of protein expression (SDS-PAGE) and oligomerization (Blue-Native PAGE) of Env_Bx08_ constructs, encoding gp120, gp140, gp140_SOSIP.R6_ and gp140_SOSIP.R6-IZ-H8_.

It is believed that vaccine immunogens should closely resemble the native trimer to improve bNAbs. Therefore, several modifications were now introduced in the three DNA constructs included in syn.gp140_mix_ to enrich for stabilized trimeric protein conformations. These are described in the experimental section and have all previously been shown to allow the efficient production of stabilized Env_JR-FL_ trimeric gene products [4]. A schematic representation of the DNA constructs is shown in Figure 3A,B. The constructs were tested for protein expression (Figure 3C), and a somewhat lower *in vitro* expression in HEK 293 cells was seen from constructs that included all the modifications (SOSIP.R6-IZ-H8). We also noted that although the IZ domain seemed to enhanced trimerization of SOSIP gp140, it also decreased to gp140 cleavage into gp120 and gp41, despite the presence of an optimal cleavage site (Figure 3C), confirming what others have reported [39,40]. Expressed gp140 with SOSIP.R6 modifications seemed to form monomers, dimers and trimeric proteins as opposed to non-modified gp140 which only appeared as monomers and dimers when analyzed by blue-native PAGE.

**Figure 4:**
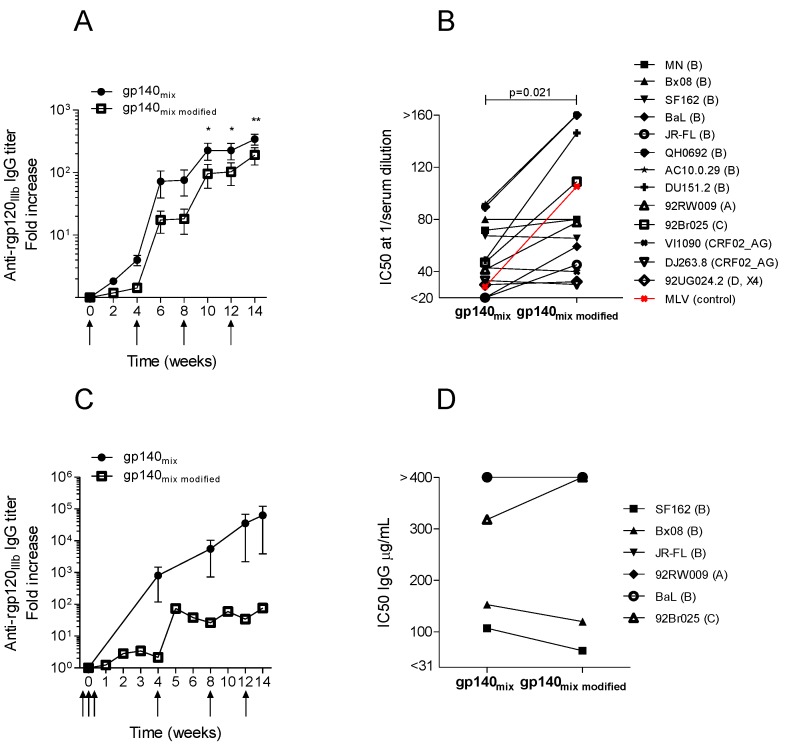
Comparison of the immune response in vaccinated guinea pigs (**A**,**B**) and rabbits (**C**,**D**) with plasmid DNA encoding syn.gp140_mix_ or syn.gp140_mix modified. _Average IgG response against recombinant gp120_IIIb_ (rgp120_IIIb_) in immunized (**A**) guinea pigs (n = 4) and (**C**) rabbits (n = 4). Immunization time points are indicated with arrows. Asterisk indicates significant difference between the two immunization groups (* *p* < 0.05, ** *p* < 0.01, two-way ANOVA). Average neutralizing activity, expressed as IC50, of (**B**) diluted guinea pig serum or (**D**) purified rabbit IgG from week 14 animal sera against pseudotype virus strains of clade A–D and CRF02_AG. Amphotropic murine leukemia virus (MLV) pseudotype virus was included as control for the non-specific activity in experiments with guinea pig serum (red). Results from syn.gp140_mix_ immunizations were derived from Figure 2.

Both guinea pigs and rabbits were immunized with the modified DNA constructs, syn.gp140_mix_ _SOSIP.R6-IZ-H8_. The guinea pigs demonstrated specific IgG after the initial immunization which was boosted upon re-immunizations; however, the modified construct, syn.gp140_mix SOSIP.R6-IZ-H8_, induced significantly lower titers of antibodies when compared to non-modified syn.gp140_mix_, as per Figure 2A (compared in Figure 4A). Interestingly, vaccination with syn.gp140_mix SOSIP.R6-IZ-H8_ generated a statistically significant higher neutralizing activity than syn.gp140_mix_ in the guinea pigs (*p* = 0.021, Wilcoxon signed rank test) despite the lower ELISA titers (Figure 4B, and Supplementary Table 1). However, the more potent neutralizing activity also included non-specific neutralization since a MLV pseudotype virus was also neutralized at high dilutions of guinea pig syn.gp140_mix SOSIP.R6-IZ-H8_ antisera. This unspecific neutralization was not seen with the non-modified syn.gp140_mix_ in Figure 2B. Immunization of rabbits with the same construct resulted in lower antibody titers for syn.gp140_mix SOSIP.R6-IZ-H8_, as compared with non-modified construct in Figure 2C (compared in Figure 4C), and similarly, as seen with guinea pig sera. Though, in the rabbit model the two constructs yielded similar neutralizing activity for the six different viruses tested (compared in Figure 4D, and Supplementary Table 1). Only two of the six viruses used could be neutralized to 50% by syn.gp140_mix SOSIP.R6-IZ-H8_ antisera at the IgG concentrations tested, and they were both clades B pseudotype virus. 

These data indicate that neutralizing activity can be improved by use of DNA vaccines encoding for modified Env immunogens, syn.gp140_mix SOSIP.R6-IZ-H8_, but the increased activity in guinea pigs is non-HIV specific. IgG was purified from a few selected guinea pig serum samples and tested in the TZMbl assay (data not shown). The guinea pig IgG displayed very low neutralizing activity. The unspecific neutralizing activity of modified constructs was only observed in immunized guinea pigs, while rabbit IgG resulting from the modified trivalent vaccine displayed similar neutralizing activity as non-modified. Since the non-modified DNA constructs indeed induced higher cross-reactive IgG titers in both animal models, and the HIV-specific immune response appeared similar for both constructs, we decided to use non-modified syn.gp140_mix_ as the vaccine in the cynomolgus macaques.

### 3.4. Immunization with the Same Optimized DNA Vaccine Induces Different Neutralizing Responses in Different Animal Models

To evaluate if neutralizing response could be translated from small animal models into non-human primates, cynomolgus macaques were immunized with the same DNA construct, non-modified syn.gp140_mix_, used in guinea pigs and rabbits, and with the same immunization regimen ranging over four months. 

**Figure 5:**
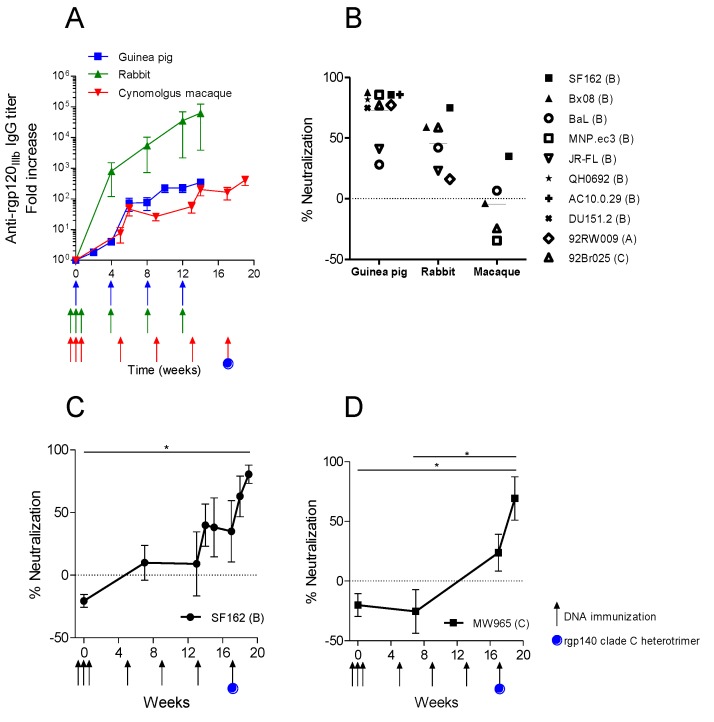
Comparison of immune response in guinea pigs, rabbits and cynomolgus macaques immunized with plasmid DNA encoding syn.gp140_mix_. (**A**) Average IgG response against rgp120_IIIb_ in immunized animals (n = 4). Immunization time points are indicated with arrows. IgG titers in rabbits and guinea pigs were derived from Figure 2A,C. (**B**) Average percent neutralization against pseudotype virus strains of clade A–C, by week 14 rabbit IgG or guinea pig sera and week 17 macaque sera. From rabbit sera, IgG was purified and used in neutralization at one fixed concentration (250 or 400 µg/mL). Sera from guinea pigs and macaques were diluted 20 and 30 times, respectively, and used in neutralization. Neutralization results of rabbit and guinea pigs were derived and recalculated from Figure 2B,D. (**C** and **D**) Macaque sera was tested for neutralization at 1/30 dilution against SF162 and MW965 viruses with the addition of a final immunization with DNA and protein at w17 (* *p* < 0.001, One-way ANOVA, Friedman’s test with Dunn’s Multiple Comparison Test).

Evaluation of gp120-specific IgG in immunized cynomolgus macaques demonstrated a response already after the initial priming immunizations; however, the antibody titers did not increase with the same magnitude as in rabbits (Figure 5A). Neutralizing capacity of antisera obtained from the immunized cynomolgus macaques was measured in the TZMbl assay against five different HIV-1 virus strains of clade B and C (Figure 5B). Percent neutralization was compared to guinea pig sera and purified rabbit IgG which had been tested against 10 and six viruses, respectively. Macaque and guinea pig sera were tested at a fixed serum dilution and rabbit IgG at a fixed concentration. Four virus strains, SF162, Bx08, BaL and 92Br025, were tested for NAbs from all three animal species. All four viruses demonstrated lower sensitivity to neutralization by macaque antisera as compared to guinea pig sera or rabbit IgG and could not be inhibited to 50% with macaque serum. The remaining virus tested with macaque sera, MNP.ec3, and was easily neutralized by guinea pig sera, but resistant to neutralization by macaque sera. 

Kinetics of neutralization of one virus, SF162, was compared between rabbit IgG and macaque sera (Supplementary Figure 2). The rabbits developed neutralizing IgG already after the second immunization at week 4, whereas neutralization in macaques developed more slowly, with a substantial increase in activity after the final immunization at week 13. However, only sera from two out of four animals reached neutralization of the virus at >50% inhibition. 

Hence, the parallel immunizations using the same DNA construct in three different animal models induced specific antibody responses in all animals, but the neutralization activity was lower in the cynomolgus macaques compared to guinea pigs and rabbits. In order to test if the immune response following the DNA immunizations could be boosted with protein, the macaques were injected with a final immunization including both syn.gp140_mix_ DNA and a clade C rgp140 heterotrimer protein. The final immunization resulted in a fast increase in antibody titers (Figure 5A) and a boost in NAbs (Figure 5C,D).

## 4. Discussion

In this study we have rationally selected, systematically optimized and evaluated HIV-1 *env* DNA vaccine constructs for immunogenicity in rabbits and guinea pigs. Our evaluation resulted in selection of trivalent gp140 vaccine (syn.gp140_mix_), encoding no modifications for stabilization of trimer formation. This construct was subsequently used for immunization of cynomolgus macaques and immune responses in the three different animal models that were compared.

An optimal DNA vaccine protocol using repeated priming injections during week 0 and i.d. electroporation was established in the rabbit model. The intensive priming resulted in faster, higher and more uniform antibody titers, likely a result of the more frequent or continued presence of the expressed immunogen, as similarly shown for T cell responses [52]. Use of intensive priming DNA vaccination with syn.gp140_mix_ resulted in immunogenicity in the macaques as well. However, compared with the rabbit model, the antibody titers and the neutralizing potency and breadth of the macaque immune response were remarkably low. The guinea pigs demonstrated a very potent immune response, despite the same increase in antibody titers as the macaques. The three different animal sera were diluted slightly differently in the comparative neutralization assay—guinea pigs 1/20 dilution, macaques 1/30, and rabbits 1/25–40 times (according to a total IgG serum level of 10 mg/mL in rabbits [53]). Still, these alterations probably do not influence the large difference seen in neutralization activity, with an average neutralization of 72% for guinea pig sera, 46% for rabbit IgG and 0% for macaque sera.

During optimization of the completely codon exchanged synthetic DNA constructs, three different aspects were considered and systematically tested in rabbits and guinea pigs. Firstly, it was evaluated whether a membrane-bound envelope product could induce a higher or broader response than its soluble form. Ideally, the final gene product from the DNA vaccine construct is a mimic of the native Env glycoproteins. Secreted soluble gp140 molecules, containing the gp120 surface glycoprotein and the ectodomain of gp41, exist in several molecular forms from transfected cells e.g. monomers, dimers, trimers, tetramers and higher molecular weight aggregates [54]. The membrane-bound gp150 product has a higher potential to mimic the native trimeric spikes [18] and to induce polyreactive antibodies that are also broadly neutralizing and targeting epitopes in the membrane proximal external region (MPER) of gp41 [55,56,57]. Moreover, expressing membrane-bound protein in the DNA-priming phase before protein boost has been suggested to give a small advantage over soluble gp140 Env proteins in terms of subsequent immune response after protein boost [10]. For these reasons, we hypothesized that the Env membrane-bound product would be superior to the secreted gp140 molecule. However, when rabbits were immunized with syn.gp140_Bx08_ or syn.gp150_Bx08_, the membrane-bound gene product did not seem to induce a higher neutralizing activity and three viruses out of six tested were actually easier to neutralize with syn.gp140_Bx08_ induced antisera. These results are indeed in agreement with our previous publication [34] in which no differences in antibody response were documented when guinea pigs were immunized with syn.gp140_Bx08_ or syn.gp150_Bx08_ constructs. The same constructs were also used to immunize rhesus macaques [34] and although syn.gp150_Bx08_ antisera showed slightly higher *in vitro* neutralizing activity than syn.gp140_Bx08_ antisera of homologous HIV-1_Bx08_, the difference did not reach statistical significance.

The second aspect considered the possibility to broaden the neutralizing response by simultaneous immunization with three different *env* genes. A polyvalent approach of administering multiple Env proteins as opposed to a monovalent Env has proven effective to broaden the Ab response in several studies including rabbits and macaques [48,49,50,51,58,59]. Nevertheless, the antigens need to be selected carefully to maximize the generated immunity. In addition to *env*_Bx08_ [60], envelope immunogens ctl21 and ctl27 were selected from individuals in whom the neutralizing capacity of serum extended to a panel of clade B R5 HIV-1 strains. We hypothesized that *env* DNA immunogens from such individuals could induce immunity against several different virus strains. Immunization of rabbits and guinea pigs with the trivalent syn.gp140_mix_, including syn.gp140_Bx08, ctl21, ctl27_, did indeed induce a response that could neutralize several different viruses of different clades, but when compared to monovalent vaccine, no increase or broadening of the neutralizing activity was achieved. This could be partly due to all three *envs* being clade B with not enough differences to induce a broader response. However, it is encouraging that *env* from only intra-clade B viruses can induce immune response against other clades. Adding more and diverse envelope genes of other clades in the DNA vaccine may further increase the broadness by either focusing the immune response to the shared conserved regions of Env while reducing the dominance of individual hypervariable regions, or simply increase the polyreactivity in an additive manner. The increased immune response observed by boosting with clade C protein/DNA mix in macaques, indicated a recall response to shared epitopes and could thus support a strategy of adding more heterologous *env* in a more polyvalent mixed vaccine strategy.

The final optimization step undertaken in regard to the DNA construct was the use of genetically modified variants of envelope genes, aiming to improve the immune responses by generating more native-like *in situ* trimers. Immunizations of rabbits with gp140 trimeric proteins with SOSIP modifications have been shown to be superior in eliciting neutralizing antibodies compared to matched monomeric gp120 protein [9,10,11]. In this study, we have engineered plasmids encoding SOSIP.R6-IZ-H8 envelope proteins for all three *env* genes used, syn.gp140_Bx08_, syn.gp140_ctl21_ and syn.gp140_ctl27_. Modified *env* constructs were mixed (syn.gp140_mix SOSIP.R6-IZ-H8_) and used for DNA immunization of rabbits and guinea pigs and compared to a non-modified mix. Immune responses however differed between the animal species. Antibodies from immunized rabbits demonstrated no difference in neutralizing activity when immunized with modified *env* or non-modified *env*, whereas sera from guinea pigs immunized with syn.gp140_mix SOSIP.R6-IZ-H8_ did generate a higher and broader neutralizing activity than syn.gp140_mix_ guinea pig antisera. But this increase in neutralizing activity of syn.gp140_mix SOSIP.R6-IZ-H8_ in guinea pigs is explained by a non-HIV specific immune response, since MLV control was also neutralized. Thus, the modified constructs seem to have induced a non-specific and broader immune response. This could not be explained by a cross-reactive antibody response, since purified IgG from guinea pig sera only demonstrated low HIV neutralizing activity (data not shown). We can only speculate that there may be a synergistic effect between the specific IgG measured in ELISA and some unspecific serum effects. If this unspecific effect was also present in immunized rabbit sera is not known since only purified rabbit IgG was tested in neutralization assays. However, purified rabbit IgG demonstrated a clear HIV-specific neutralizing effect whereas purified guinea pig IgG did not. Several explanations may be given as to why the immunization experiments described in this study cannot confirm that SOSIP-modifications offer an advantage in the rabbit model in induction of NAbs. Our study differs in many aspects compared to other SOSIP studies using rabbits. Previous studies include SOSIP-modified recombinant glycoproteins [9,10,11], whereas we used SOSIP-modified DNA constructs expressed via DNA vaccination *in vivo*. When producing SOSIP gp140 recombinant *in vitro*, it is easier to control and ensure precursor cleavage, an aspect that might contribute to the favorable antigenic and immunogenic properties of SOSIP gp140. Furthermore, it is possibly to purify gp140 trimer proteins out of the mixtures of monomers, dimers, trimers and aggregates that are usually formed. Uncleaved and non-trimeric gp140 forms produced *in vivo* upon DNA immunization might distract the immune response from cleaved gp140 trimers that better recapitulate the antigenic structure of the native Env spike. Finally, all clinical isolates may not benefit from the same SOSIP mutations deduced from the JRFL strain equally well [4].

In order to accelerate the vaccine design process, model systems are important to screen candidate immunogens such as those from selected patient HIV-1 isolates. The model used however is of great importance when assessing immunogenicity, and advantages and drawbacks with each animal model should be considered as well as knowledge of potential antibody gene repertoires and gene usage frequencies [3]. When evaluating a potential human vaccine candidate the most reliable animal model today is the macaque, which shares the pathogenic effects of HIV-1 seen in humans. However, the ethical and financial concerns regarding macaque experiments makes it necessary to assess immunogens in smaller animal models before they can be used in the macaque model. In the present study, optimization of immunogens in rabbits differed somewhat from guinea pigs, and did not automatically translate well into cynomolgus macaques. One conclusion from this is that it is important to select a relevant animal model for optimal selection of immunogens, followed by evaluation of dose, delivery route, method and specific immune response generated in an iterative process. Even with our rationally selected and optimized DNA immunogens, higher antibody potency seemed necessary in the macaques. This could be achieved by using a purified clade C heterologous trimeric protein as a boost in which the adjuvant was in fact the DNA vaccine mixed with the protein that boosted NAb to both clade B and C strains. 

Among the existing models, mice have not been used extensively for testing of HIV *env* DNA vaccines due to the Rev dependence and the poor expression of these genes in mice. However, this problem can be overcome by codon-optimization of genes [18,35], which has made it possible to achieve comparable immune responses in mice and macaques [61]. However, rodents, including mice, lack the ability to produce antibodies with long CDR3 loops [24]. Since these loops are important features of several known broadly neutralizing antibodies [3,26,27,28,29,30], the rodent models have a clear disadvantage when screening for immunogenicity. The rabbit model, being a lagomorph and a larger animal, may more closely resemble the macaque model and still maintain the advantages of being less expensive, easy to handle and with large blood volumes to work with. Rabbits may also have an advantage over guinea pigs in generating antibodies, seen after electroporation with an HIV DNA vaccine [21]. This might explain the low potency of guinea pig antibodies we noticed when we purified IgG from a few serum samples with unspecific neutralizing response (data not shown). Thus, the rabbit is a favored model for test of immunogenicity and screening of vaccine candidates, although the model does not guarantee an equal response or protective efficacy in the macaque model. Moreover, even the macaque model may prove not to adequately predict the ability of a vaccine to generate bNAbs and show efficacy in humans. As a consequence, it could prove necessary to actually use macaques or even humans in the screening for optimal HIV-1 Env immunogens eliciting bNAbs and use small animal models primarily to ensure immunogenicity of the DNA constructs.

## 5. Conclusions

Rational selection of envelope genes and thorough screening concluded that a trivalent mix of non-modified gp140 genes is optimal to induce high and broad NAb in the preferred rabbit model. However, this optimization differed from guinea pigs and did not directly translate into cynomolgus macaques. This suggests species-specific differences in the quality of immune response to HIV-1 *env* DNA and emphasizes the importance of choosing the correct animal model when screening for future vaccine constructs. 

## Supplementary Files

Supplementary File 1
