# Supplementary material for: Optimization of HIV-1 Envelope DNA Vaccine Candidates within Three Different Animal Models, Guinea Pigs, Rabbits and Cynomolgus Macaques
Source: Vaccines (Basel). 2013 Jul 19;1(3):305–27. doi: 10.3390/vaccines1030305 (PMC4494233; doi:10.3390/vaccines1030305)
Supplement: Supplementary File 1 [file vaccines-01-00305-s001.pdf]

Supplementary Material

Figure S1. Comparison of the immune responses in rabbits vaccinated with standard DNA immunization regimen (n = 3), DNA immunization with "intensive" priming (n = 2) and protein immunization (n = 3). DNA immunization contained the same *env* gp140Bx08 and the protein immunization was with rgp120IIIb in CAF01 adjuvant. Immunization time points are indicated with arrows. Specific IgG response was measured against strain IIIb gp120.

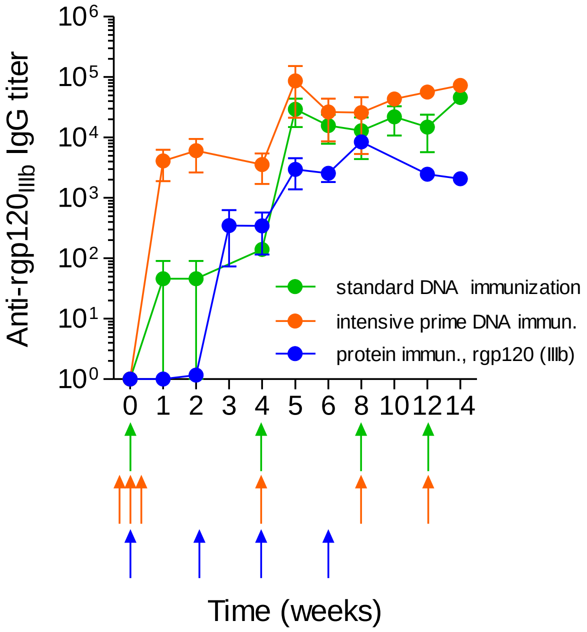

Figure S2. Average neutralization kinetics of rabbit IgG (green) and macaque sera (red) of virus strain SF162. Rabbit IgG was tested at 250 µg/mL and macaque sera was diluted 30 times. Both groups of animals included 4 animals, immunized with the same plasmid DNA encoding syn.gp140$_{mix}$. Immunization time points are indicated with arrows.

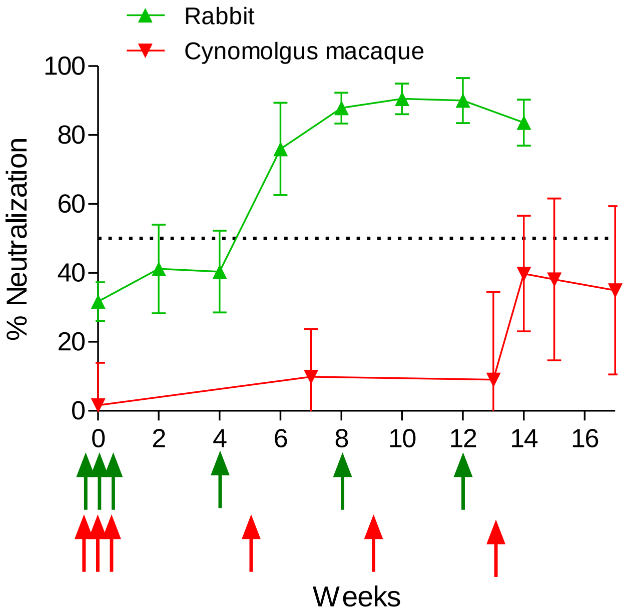

Table S1. Neutralizing activity in serum from immunized guinea pigs and rabbits. Pre-immune sera and 14 weeks post vaccination was tested. Darker color corresponds to higher neutralizing activity. For guinea pigs reciprocal titers correspond to 1/dilution of serum giving 50% inhibitory concentration (IC50) are described. For rabbits purified IgG corresponding to 50% inhibitory concentration (IC50) are described.

															Tier 2		
Animal	*Vaccine construct*	*Animal ID*	*Sample*	*MLV*	*B*	*B*	*B*	*B*	*A*	*C*	*CRF02_AG*	*CRF02_AG*	*D; (X4)*	*B*	*B*	*C*	*Subtype*
					Bx08	*SF162*	*BaL*	*JR-FL*	*92RW009*	*92Br025*	*VI1090*	*DJ263.8*	*92UG024.2*	*QH0692*	*AC10.0.29*	*DU151.2*	*Strain*
Guinea pigs	gp140×08	5622	Preimmune	74	<20	<20	<20	<20	<20	<20	<20	<20	<20	<20	<20	nd	
		5623		34	<20	<20	<20	<20	<20	<20	<20	<20	<20	<20	<20	<20	
		5624		26	<20	<20	<20	<20	<20	<20	<20	<20	<20	<20	<20	<20	
		5625		33	<20	<20	<20	<20	<20	24	<20	<20	<20	<20	23	<20	
	Gp140Mix	5634		26	<20	<20	<20	<20	<20	<20	<20	<20	<20	21	<20	<20	
		5635		24	<20	<20	<20	<20	<20	<20	<20	<20	<20	24	<20	<20	
		5636		22	<20	<20	<20	<20	<20	21	<20	27	<20	<20	23	<20	
		5637		21	<20	<20	<20	<20	<20	<20	<20	<20	<20	<20	<20	<20	
	Gp140Mix SOSIP-R6-IZ-H8	6460		30	30	36	38	23	<20	25	<20	<20	<20	28	36	35	
		6461		26	41	24	32	<20	<20	25	<20	<20	<20	29	32	29	
		6462		28	29	<20	37	23	23	27	<20	<20	<20	31	33	26	
		6463		29	<20	<20	23	<20	21	27	<20	<20	<20	30	31	26	
	Gp140B×08	5622	Week 14 post vaccination	26	>80	27	<20	<20	37	58	38	24	32	60	76	62	
		5623		<20	>80	>80	<20	25	69	35	38	30	27	127	>160	>160	
		5624		<20	74	<20	<20	<20	35	28	28	23	23	39	52	37	
		5625		22	>80	<20	<20	<20	31	37	26	25	28	61	54	55	
	Gp140Mix	5634		34	>80	>80	<20	<20	38	46	50	35	33	94	95	40	
		5635		28	>80	>80	<20	<20	30	36	34	35	23	68	72	42	
		5636		32	>80	>80	<20	<20	59	74	63	39	38	134	151	79	
		5637		<20	>80	30	<20	<20	39	32	25	24	26	63	49	34	
	Gp140Mix SOSIP-R6-IZ-H8	6460		70	>80	55	68	40	132	>160	56	36	40	>160	>160	>160	
		6461		>160	>80	57	45	40	86	>160	39	34	35	>160	>160	138	
		6462		117	>80	>80	58	55	56	76	38	31	29	>160	>160	>160	
		6463		74	>80	70	66	46	37	0	27	<20	25	>160	>160	127	

Table S2. *Cont.*

Animal	*Vaccine construct*	*Animal ID*	*Sample*	*B* SF162	*B* Bx08	*B* BaL	*B* JR-FL	*C* 92Br025	*A* 92RW009	Subtype Strain
Rabbits	gp140Bx08	116036	Preimmune	>250	>250	n. d.	n. d.	n. d.	n. d.	
		116157		>250	>250	n. d.	n. d.	n. d.	n. d.	
		116064		>250	>250	n. d.	n. d.	n. d.	n. d.	
		116069		>250	>250	n. d.	n. d.	n. d.	n. d.	
	gp150Bx08	116087		>250	>250	n. d.	n. d.	n. d.	n. d.	
		116115		>250	>250	n. d.	n. d.	n. d.	n. d.	
		116126		>250	>250	n. d.	n. d.	n. d.	n. d.	
		116130		>250	>250	n. d.	n. d.	n. d.	n. d.	
	gp140Mix	134040		>250	>250	n. d.	n. d.	n. d.	n. d.	
		134114		>250	>250	n. d.	n. d.	n. d.	n. d.	
		134126		>250	>250	n. d.	n. d.	n. d.	n. d.	
		134132		>250	>250	n. d.	n. d.	n. d.	n. d.	
	gp140Mix SOSIP-R6-IZ-H8	946369		>250	>250	n. d.	n. d.	n. d.	n. d.	
		946387		>250	>250	n. d.	n. d.	n. d.	n. d.	
		946376		>250	>250	n. d.	n. d.	n. d.	n. d.	
		946370		>250	>250	n. d.	n. d.	n. d.	n. d.	
	gp140Bx08	116036	Week 14 post vaccination	<31	35	239	>400	>400	>400	
		116157		<31	174	>400	>400	343	>400	
		116064		58	143	393	>400	>400	>400	
		116069		<31	<31	154	>400	370	>400	
	gp150Bx08	116087		197	>250	>400	>400	391	>400	
		116115		72	<31	180	>400	273	>400	
		116126		<31	36	>400	>400	373	>400	
		116130		129	148	>400	>400	>400	>400	
	gp140Mix	134040		102	84	>400	>400	309	>400	
		134114		170	193	>400	>400	329	>400	
		134126		110	218	>400	>400	307	>400	
		134132		<31	81	>400	>400	323	>400	
	gp140Mix SOSIP-R6-IZ-H8	946369		128	>250	>400	>400	367	>400	
		946387		55	<31	>400	>400	>400	>400	
		946376		31	82	>400	>400	369		
		946370		64	136	>400<br>>400<br>>400	>400	374		
